# Device-based therapy for decompensated heart failure: An updated review of devices in development based on the DRI_2_P_2_S classification

**DOI:** 10.3389/fcvm.2022.962839

**Published:** 2022-09-21

**Authors:** Cristiano de Oliveira Cardoso, Abdelmotagaly Elgalad, Ke Li, Emerson C. Perin

**Affiliations:** ^1^Center for Preclinical Surgical and Interventional Research, Texas Heart Institute, Houston, TX, United States; ^2^Center for Clinical Research, Texas Heart Institute, Houston, TX, United States

**Keywords:** heart failure, acute heart failure, devices, cardiorenal syndrome, treatment

## Abstract

Congestive heart failure (HF) is a devastating disease leading to prolonged hospitalization, high morbidity and mortality rates, and increased costs. Well-established treatments for decompensated or unstable patients include medications and mechanical cardiac support devices. For acute HF decompensation, new devices are being developed to help relieve symptoms and recover heart and renal function in these patients. A recent device-based classification scheme, collectively classified as DRI_2_P_2_S, has been proposed to better describe these new device-based therapies based on their mechanism: dilators (increase venous capacitance), removers (direct removal of sodium and water), inotropes (increase left ventricular contractility), interstitials (accelerate removal of lymph), pushers (increase renal arterial pressure), pullers (decrease renal venous pressure), and selective (selective intrarenal drug infusion). In this review, we describe the new class of medical devices with the most current results reported in preclinical models and clinical trials.

## Introduction

According to the universal definition, “heart failure (HF) is defined as a clinical syndrome with symptoms and/or signs caused by a structural and/or functional cardiac abnormality and corroborated by elevated natriuretic peptide levels and/or objective evidence of pulmonary or systemic congestion” (1). In addition to this broad description, several classification systems for HF can be applied that incorporate stages of risk, the presence of symptoms, the etiology, and the assessment of left ventricular function.

Chronic HF affects about 2% of the adult population worldwide. However, its prevalence is age-dependent, ranging from less than 2% in people < 60 years old to more than 10% in those older than 75 years (2). According to recent updated data from the American Heart Association, about 6.2 million Americans ≥ 20 years of age were diagnosed with HF between 2013 and 2016 (3). Projections show that the prevalence of HF in the United States will increase 46% from 2012 to 2030, resulting in more than 8 million people with HF (4).

A wide variety of drugs, devices, and procedures are available to improve survival and functional class in patients with HF (5–7). Patients with mild to moderately decompensated HF can usually be stabilized with medical treatment. For those who are extremely ill and unstable (8), various mechanical cardiac support devices can be used to attempt stabilization or as a bridge to transplantation. However, therapeutic options are limited for patients at an intermediate stage, whose HF cannot be controlled with medication but who are not ill enough to benefit from mechanical cardiac support (9).

New device-based treatments are being developed to treat specific pathways in patients with decompensated HF. Although the physiopathology is complex, patients with acute decompensated heart failure (ADHF) (10) present with decreased cardiac contractility and low cardiac output. Low blood pressure leads to deficient perfusion of the organs, which activates the renin-angiotensin-aldosterone system, vasopressin release, and upregulation of the sympathetic nervous system. The neurohormonal changes decrease renal artery pressure and renal blood flow, promoting an increase in sodium and water retention. Fluid overload increases central venous pressures, resulting in systemic congestion and elevated abdominal pressure. Intraabdominal hypertension causes impairment of renal function (11). In addition, the swollen intestine caused by systemic congestion results in poorer absorption of diuretics (12). Rosenblum et al. (13) have proposed a new classification strategy based on seven categories for these devices that act on different mechanisms involved in the pathophysiology of HF. Denoted by the acronym DRI_2_P_2_S, the classification scheme categorizes the devices according to their mechanism and suggested indication. Table 1 summarizes the new medical device classification and mechanisms.

**TABLE 1:** DRI_2_P_2_S classification scheme for device-based therapy for heart failure.

Classification scheme	Mechanism of action	Device-based approach
Dilators (D)	Increases venous capacitance	Splanchnic nerve modulation
Removers (R)	Removes sodium and/or water directly	AlfaPump, Reprieve System
Inotropes (I_1_)	Improves left ventricular contractility	Cardionomic, NeuroTronik
Interstitial (I_2_)	Accelerates lymph removal	WhiteSwell
Pushers (P_1_)	Increases renal arterial pressure	Reitan catheter pump, Aortix, Second Heart Assist
Pullers (P_2_)	Decreases renal venous pressure	preCardia, Doraya catheter, transcatheter renal decongestion system
Selective (S)	Infuses vasodilator drugs selectively *via* the infrarenal artery	Benephit catheter

Modified from Rosenblum et al. (13).

## Dilators (D–increase venous capacitance)

Around 30% of the blood volume circulates in the arterial circulation, and the rest is confined to the venous system. Because of its large capacitance, the abdominal venous system is the main reservoir for blood volume in the body. In addition, the abdominal venous reservoir responds to sympathetic stimuli by promoting vasoconstriction and shifting fluid from the abdominal system into the circulation. In patients with HF, the shifted volume overloads and worsens peripheral congestion, increases cardiac venous return, and raises cardiopulmonary filling pressures, thus worsening pulmonary congestion.

Vasodilators (nitroprusside and nitrates) dilate venous and arterial vessels. Nitrates act mainly on peripheral veins, whereas nitroprusside affects the arterial and venous systems. Vasodilators reduce the venous return, resulting in less congestion, lower afterload, and a consequent relief of symptoms. Recently, splanchnic nerve modulation has been proposed for treating congestive HF (14). The concept underlying splanchnic nerve modulation is to block the great splanchnic nerve, which carries visceral sympathetic and sensory fibers. The splanchnic nerves, located on both sides of the spine, arise from the sympathetic thoracic trunk to innervate the abdomen. Consequently, a splanchnic nerve block would reduce the response to the abdominal reservoir to the sympathetic tonus, thus reducing the shifting of blood from its cavity to the circulation and decreasing cardiopulmonary filling pressures. Splanchnic nerve block is achieved by using a percutaneous approach under fluoroscopic guidance. First, a spinal needle is positioned to the anterolateral edge of the thoracolumbar spine at the T11–12 level, and then a local anesthetic (lidocaine or ropivacaine) is injected to temporarily block the nerve (unilateral or bilateral).

Two studies (15) have tested this concept in clinical practice. The Splanchnic HF-1 (16, 17) and Splanchnic-HF-2 (18), both small clinical studies, evaluated the physiologic effects of splanchnic nerve block in patients with acute and chronic HF, respectively. Splanchnic HF-1 prospectively assessed 11 patients with HF who had New York Heart Association class III/IV symptoms, reduced ejection fraction, and a pulmonary capillary wedge pressure (PCWP) > 15 mmHg (> 12 mmHg if on inotropes) on baseline right heart catheterization. This first-in-human, proof-of-concept study showed that splanchnic nerve block could reduce mean pulmonary arterial pressure, mean arterial pressure, and PCWP, resulting in an increase in cardiac index after the intervention. The Splanchnic-HF-2 trial tested the hypothesis that splanchnic nerve blockade would attenuate the increase in exercise-induced cardiac filling pressures in patients with chronic HF with reduced ejection fraction. In this prospective, open-label, single-arm trial, 15 patients with chronic HF and elevated PCWP underwent exercise testing before and after nerve block with ropivacaine. The findings showed that splanchnic nerve block reduced resting and exercise-induced pulmonary arterial and wedge pressure with favorable effects on cardiac output and exercise capacity, results similar to the Splanchnic HF-1 trial.

Although procedural complications (14) (pneumothorax, chylothorax, bowel perforation, vascular damage, and others) and physiologic changes (diarrhea, orthostatic hypotension, nausea, and vomiting) can occur with nerve block, the procedure was well tolerated and without significant complications in both studies.

## Removers (R–direct removal of sodium and water)

Patients with HF have an overload of water and sodium in the extracellular space due to the physiologic and adaptative changes of the failing heart. Although diuretics play an essential role in fluid removal and symptom relief, diuretic resistance may render them non-effective in clinical practice (12).

Water and sodium can be removed from the body *via* ultrafiltration (19). Previous studies have shown that ultrafiltration (20, 21) with either aquapheresis (22) or peritoneal dialysis (23, 24) can be an alternative to diuretics in controlling fluid overload in patients with congestive HF. Because classic ultrafiltration is not the focus of this review, only the newer devices are discussed.

**FIGURE 1:**
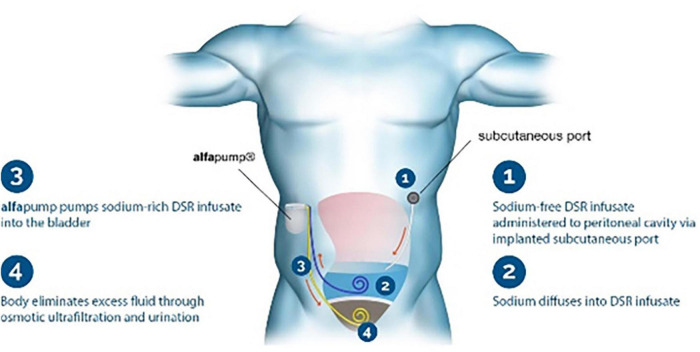
Remover device. The Alfapump DSR system for sodium removal. Image used with permission from Sequana Medical.

New devices called removers have been used to help treat volume overload in decompensated patients. One of the new devices—the alfapump DSR^®^ (Sequana Medical NV, Belgium)—is implanted subcutaneously in the abdomen and automatically and continuously moves fluid from the abdominal cavity to the bladder, where it is excreted from the body (25). *Via* a surgically implanted port, sodium-free DSR infusate is delivered into the peritoneal cavity; this approach allows for flexible dosing to remove the desired amount of sodium. The DSR infusate remains for a pre-determined time, and it and the extracted sodium are pumped to the bladder and eliminated in the urine (Figure 1). In a proof-of-concept study in pigs, the device removed 4.1 ± 0.4 g of sodium from the body in 2 h, with no significant changes in other electrolytes. The ongoing clinical trial SAHARA (NCT04882358) will enroll 24 patients with congestive HF and diuretic resistance. In an interim analysis of six patients in the trial (26), the new device was safe and tolerable, and treated patients showed a mean weight loss of 6 kg compared to baseline and a 30% reduction in NT-proBNP; the estimated glomerular filtration rate (eGFR) was not significantly affected.

The Reprieve System™ (Reprieve Cardiovascular, Milford, MA, USA) is another new decongestion device for improving outcomes for patients with ADHF. The goal in using the Reprieve device is to achieve a target fluid balance. The system comprises a peripheral or central infusion port and a Foley urinary catheter that is connected to an external console. The Reprieve System constantly measures the patient’s urine output and infuses a volume of hydration fluid sufficient to maintain a set fluid-balance rate. Two clinical trials provided results on the use of this technology. Target-1 and Target-2 (NCT03897842) trials evaluated the device in 19 patients with congestive HF and preexisting impaired renal function, respectively (27). The findings showed the Reprieve System was safe and tolerable in all patients. In addition, patients treated with the Reprieve System lost weight during hospitalization (–3 kg, *p* < 0.001) and showed improved renal function (baseline creatinine levels of 1.45 ± 0.4 vs. 1.26 ± 0.4 mg/dl at end of therapy, *p* = 0.0002) and decreased CVP (from 15.5 ± 5.3 mmHg at baseline to 12.8 ± 4.8 mmHg at end of therapy; *p* = 0.02).

## Inotropes (I_2_–increase left ventricular contractility)

The heart is innervated by the cardiac plexus of nerves situated at its base. Cardiac branches are derived from both the sympathetic and parasympathetic nervous systems. In a dog model (28), cardiac plexus stimulation increased cardiac contractility and mean arterial pressure with no changes in heart rate. This concept has been tested in humans with two different devices.

**FIGURE 2:**
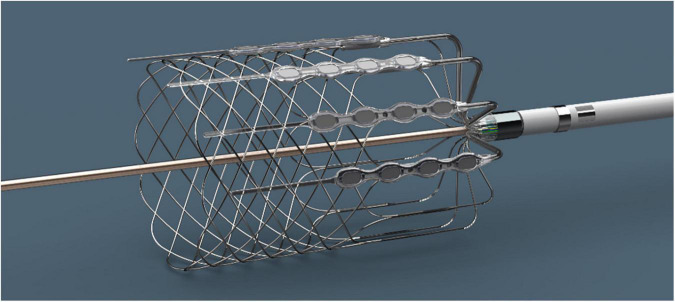
Inotrope device. The Cardionomic Cardiac Pulmonary Nerve Stimulation (CPNS). Image used with permission from Cardionomic, Inc.

The catheter-based cardiopulmonary nerve stimulation (CPNS, Cardionomic Inc., New Brighton, MN) is a 16-French catheter device that is inserted percutaneously through the right jugular vein and positioned in the right pulmonary artery (Figure 2). The CPNS is used to provide endovascular stimulation for up to 5 days in patients with ADHF. In an ongoing clinical trial of CPNS (NCT04814134), initial results (29) in 7 patients showed no adverse events. CPNS therapy increased heart contractility (LV dP/dt max) by 58%, left ventricular relaxation (LV dP/dt min) by 11%, arterial pulse pressure by 20%, and mean arterial pressure by 7%. No significant changes were observed in heart rate. Target enrollment for the trial is 50 patients for evaluating long-term impact.

Using a similar concept, the NeuroTronik CANS Therapy™ System (NCT03169803, NCT02880683, and NCT03542123) is a purpose-built electrical stimulation catheter placed percutaneously in the left brachiocephalic vein *via* left subclavian vein access. The neurostimulator is then connected to the catheter and is used to deliver autonomic nerve stimulation therapy for up to 96 h. At the 2019 Transcatheter Cardiovascular Therapeutics (TCT) symposium (30), investigators presented the initial results of a single-arm study in 12 patients with congestive HF (at least two signs and symptoms) and an ejection fraction < 40%. NeuroTronik therapy improved cardiac index (+ 22%) and decreased PCWP (–28%) and systemic vascular resistance (–22%). No significant changes were observed in cardiac rate.

## Interstitial (I_2_–accelerate removal of lymph)

**FIGURE 3:**
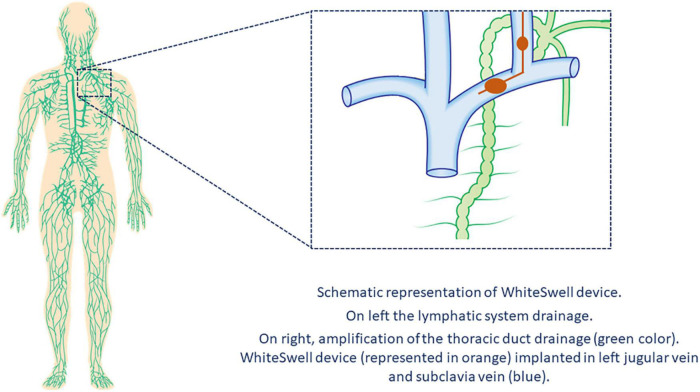
Interstitial device. The WhiteSwell device.

In healthy people, excess liquid in the interstitial space is removed through the lymphatic system, which collects fluid in these spaces and returns it to the veins *via* the thoracic and lymphatic ducts. As a consequence of HF, lymphatic drainage of edema in the periphery, abdominal organs, and lungs is reduced (31). The WhiteSwell™ therapy system (WhiteSwell, Ireland) is a new device designed to accomplish complete decongestion while preserving renal function. The device comprises a multi-lumen catheter with two compliant balloons of low-durometer urethane. The catheter balloons are positioned across the bifurcation of the jugular and innominate veins and isolate the thoracic duct outflow when inflated. The system produces a decrease in the local pressure (between both inflated balloons) and reduces venous pressures in the thoracic duct outflow area, thus facilitating drainage of the thoracic duct (Figure 3).

In a sheep model of HF, Abraham et al. (32) conducted a proof-of-concept study of the WhiteSwell device. Dilated cardiomyopathy was created by serial coronary embolization, and fluid overload was used to create decompensated HF with pulmonary congestion. The WhiteSwell device was activated for up to 3 h in the four treated sheep; 3 served as controls. Extravascular lung water volume was decreased in treated sheep as compared to controls. The authors also reported a case of an 82-year-old woman who is part of an ongoing clinical trial (NCT02863796) on the safety and feasibility of the WhiteSwell device. She had presented with HF with preserved ejection fraction, hypertension, chronic atrial fibrillation (treated with novel oral anticoagulants), chronic renal failure, and severe pulmonary hypertension. After receiving standard clinical therapy, she was treated with the WhiteSwell device, which was introduced *via* the left internal jugular vein under fluoroscopy guidance. Device treatment significantly increased urine output rate and reduced central venous pressure during the therapy. Results of the ongoing trial have not been published.

## Pushers (P1–increase renal arterial pressure)

**FIGURE 4:**
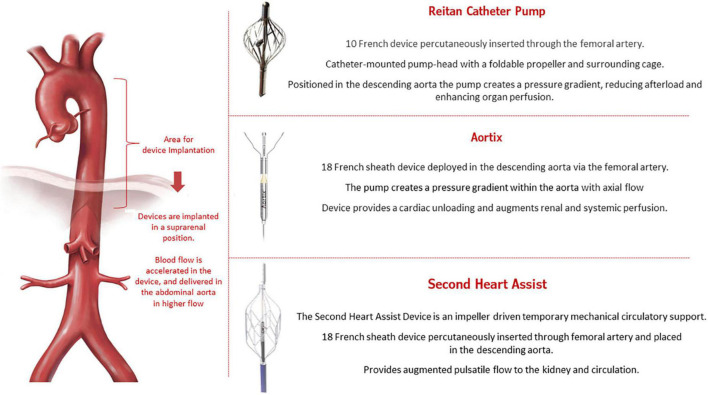
Pusher devices for heart failure.

The close interaction between the kidney and heart in patients with HF has been called cardiorenal syndrome (CRS) (33, 34). CRS is a complex entity that involves both organs and neurohormonal mechanisms, with a physiopathology characterized by low cardiac output leading to decreased renal perfusion. New devices are being developed to increase renal output in HF. These devices, called pushers, are intended to be temporarily implanted in the descending aorta above the renal arteries and are supposed to increase the flow toward the renal arteries to promote better perfusion in the kidneys. In addition, pushers can also decrease left ventricle afterload. Figure 4 illustrates the concept of the new pusher devices: Reitan catheter (35), Aortix (36), and Second Heart Assist.

The Reitan catheter pump (Cardiobridge, Germany) was first tested in nine patients requiring mechanical circulatory support during complex percutaneous coronary intervention (PCI) (37). The propeller was set to rotate around 10,000 rpm, promoting a gradient between the radial-femoral pressure of around 10 mmHg. The main finding was that using the device reduced creatine levels by an average of 11 ± 8 μmol/l (*P* = 0.004) from before to after the procedure. In a larger prospective, observational study (38), 18 patients admitted with decompensated HF, an ejection fraction < 30%, and a cardiac index < 2.1 L/min/m^2^ who were in need of inotropic/mechanical support received the device-based therapy. The mean running time of the Reitan device was 18.3 h, and treated patients showed an increase in diuresis, renal function, and cardiac index.

The Aortix (Procyrion, Inc., Houston, TX, USA) is an axial-flow pump that is positioned in the aorta to provide short-term hemodynamic support. The device functions to promote a higher pressure in the distal abdominal aorta (39, 40). The first-in-human study was conducted by Vora et al. (41) and enrolled six patients with renal dysfunction to receive the device during high-risk PCI. Aortix was implanted for a mean time of 70 min with no severe complications. In addition, device support improved urine output (10-fold) and eGFR (mean increase, 6.95 ± 8.09 mL/min). Another case in which this device was successfully implanted was presented at the 2021 TCT symposium (42).

Second Heart Assist (Second Heart Assist, Salt Lake City, UT, USA) is another investigational device that promotes circulatory support for patients with decompensated HF and CRS who are at high-risk for PCI. This stent-based impeller pump is percutaneously inserted (*via* the femoral artery) and enables better flow to the kidneys. No clinical data have been published, but the preclinical data are encouraging.

Although all of the devices in this category have the potential risk for hemolysis, infection, and thromboembolic complications, no serious adverse events have been reported for any of them. However, it is important to be cautious about the potential risk that any invasive device confers in terms of hemocompatibility complications and possible infection. Before being tested in a clinical setting, new devices should be examined in depth for these potential hazards.

## Pullers (P_2_–decrease renal venous pressure)

Devices in the pullers category are used to reduce cardiac volume overload and filling pressures at the superior/inferior vena cava (IVC) or venous congestion at the abdominal cavity.

**FIGURE 5:**
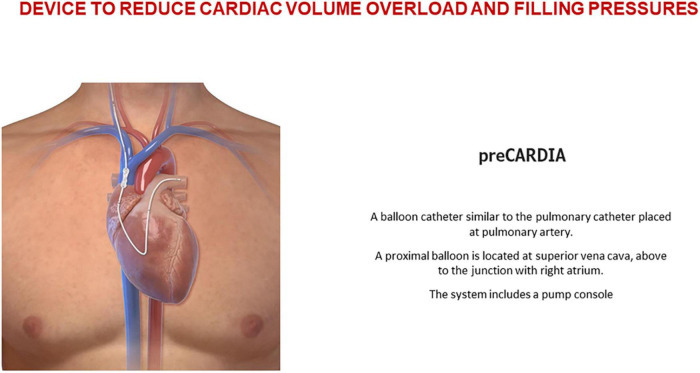
Puller device for heart failure. The preCardia. Image used with permission from Abiomed.

The preCARDIA balloon catheter device (Abiomed, Danvers, MA, USA) is placed in the superior vena cava (SVC) for intermittent occlusion. Controlled by a pump, the preCARDIA device unloads the heart, decreasing its filling pressures and helping to achieve decongestion in HF patients (Figure 5). In a proof-of-concept study, Kapur et al. (43) demonstrated that transient occlusion of the SVC reduces cardiac filling pressures without significant changes in cardiac output and blood pressure. In their study, eight patients with decompensated HF underwent intermittent balloon occlusion in the SVC; the procedure was well-tolerated and reduced cardiac filling pressure. The VENUS-HF trial (44) (NCT03836079), an early feasibility study, was a multicenter, prospective, single-arm trial of patients who were treated for 12–24 h with the preCARDIA system. Compared with baseline values, right atrial pressure decreased by 34%, PCWP declined by 27%, and urine output and net fluid balance increased by 130 and 156%, respectively. Similarly, intermittent occlusion of the IVC has also effectively reduced cardiac filling pressures (45, 46).

As a result of systemic congestion, patients with HF have elevated central venous and intraabdominal pressure (IAP). Moreover, venous congestion is emerging as an important cause of renal dysfunction in patients with CRS, and the term “congestive nephropathy” has been proposed for this new concept (47). Animal studies (48–50) have shown that an elevated IAP is related to renal dysfunction. In a study of pigs with induced pneumoperitoneum, Toens et al. (51) demonstrated that IAP > 40 cmH_2_O led to renal dysfunction caused by tubular epithelial necrosis. In clinical medicine, an elevated IAP (≥ 8 mmHg) has also been correlated to renal dysfunction (52) and to an increase in 1-year mortality when IAP > 12 mmHg for more than 72 h after admission (53).

Different devices can be placed below, above, or even within the kidney to reduce kidney congestion. For example, the Doraya catheter (Revamp Medical Ltd., 5 Mefi, Netania, Israel) is a manageable flow-reducing device implanted in the infrarenal IVC for up to 12 h. The temporary mechanical obstruction of flow reduces central venous pressure, renal afterload, and venous returns, thus relieving congestion on the heart, lungs, and kidneys. Two published cases (54) have demonstrated that the Doraya device could promote cardiac decongestion and improve diuretics resistance. A clinical trial of the Doraya catheter for the treatment of acute HF (AHF) (NCT03234647) has been completed, but no published results are available. Another device called Transcatheter Renal Venous Decongestion™ (TRVD) System (Magenta Medical, Kadima, Israel) is designed to reduce the pressure in both renal veins through a catheter-based approach by using an axial-flow pump-head positioned in the IVC. Two sealing elements are placed above and below the kidneys to compartmentalize the renal segment of the IVC and allow selective reduction of renal venous pressures. The TRVD System is intended to be placed shortly after hospital admission to mechanically unload the kidneys for 1–3 days. The first-in-human trial (NCT03621436) tested the concept (55) in 13 patients with HF and low ejection fraction. After TRVD therapy, renal venous pressure decreased (from 19.2 ± 4.1 to 10.5 ± 3.3 mmHg, *p* < 0.00001) as did right atrial pressure. The clinical trial is completed, but no results are available.

## Selective (S–selective intrarenal drug delivery)

Decreased renal perfusion and vasoconstriction trigger tubular hypoxia and are part of the complex mechanism of kidney function impairment in patients with HF (56). Vasodilator drugs are used to prevent renal vasoconstriction and reduce kidney dysfunction. Although clinical trials have shown that low doses of dopamine or nesiritide (57) did not improve renal function in HF patients, the concept is still being studied in different clinical scenarios. Teirstein et al. (58) tested a selective infusion of fenoldopam (a dopamine 1 receptor agonist that decreases peripheral vascular resistance primarily in renal capillary beds) into the kidneys with a Benephit catheter (Angiodynamics, Latham, NY) during coronary angiography. The Benephit is a bifurcated catheter percutaneously inserted through the femoral artery for selective infusion in both renal arteries. In a randomized, open-label, partial crossover design trial in 33 patients, the use of the Benephit catheter for the selective renal infusion of fenoldopam was safe and produced some benefit in renal function. In a post-market registry (59), the use of the Benephit catheter system to infuse fenoldopam was examined in 501 patients at high-risk for contrast-induced nephropathy during coronary or peripheral angiography/intervention or cardiovascular surgery. The study showed this approach was safe and resulted in a lower incidence of contrast-induced nephropathy than predicted by the Mehran score. Although this intra-renal selective infusion therapy has demonstrated some benefit during coronary interventions, it has not been tested in the specific clinical scenario of HF; therefore, comments and preliminary results should be cautiously considered.

## Discussion

AHF, defined as the rapid development of new symptoms and signs of HF, can be differentiated into ADHF and *de novo* AHF (60). AHF is a presentation caused by an acute heart injury (e.g., myocardial infarction or myocarditis), whereas ADHF is commonly seen in patients with a history of HF who have an imbalance due either to volume redistribution or to overload. The classic clinical presentations of ADHF are signs and symptoms of congestion and volume overload (dyspnea, orthopnea, lower limb edema, ascites). Accounting for most cases of acute decompensation, ADHF is seen often in older patients and has higher mortality rates and more comorbidities than AHF (61).

In addition to the underlying mechanisms of the acute decompensation (disease progression or secondary factor triggering the decompensation), patients with ADHF have changes in myocardial heart contraction and pulmonary function as well as renal dysfunction and intraabdominal changes. Combined, these factors make HF a complex disease to manage, and compensating for these changes with medications is challenging. A multidisciplinary HF management program is mandatory for evaluating patients; this approach ensures that the correct investigations are conducted and that an accurate diagnosis is made. Then, the appropriate evidence-based therapy may be initiated to treat the mechanism that is identified as the primary cause of the acute decompensation. A team approach for HF care has been demonstrated to reduce mortality and hospitalizations in high-risk patients (62), and these strategies play an important role in HF treatment. In addition, new approaches such as telemedicine support and wearable devices can be integrated into the treatment regimen. The new devices described in this review are not meant to replace current HF treatment. Few treatment options are available for the group of patients in whom decompensation cannot be treated with medications but who are not unstable enough to qualify for mechanical support; these new devices may offer an alternative therapeutic option to fill that gap. It is important to emphasize that the devices do not specifically address the mechanisms leading to decompensation. Rather, they support the heart and kidneys to improve preload, afterload, and renal perfusion and function. It is expected that the new devices will help to reverse the decompensation episode because each device acts specifically on the pathway that is exacerbating the decompensation. Thus, patients will benefit by more precisely addressing the mechanisms causing the symptoms during a decompensation episode. If needed, specific heart and kidney support may be possible with the new-device based therapy.

HF has an enormous impact on quality of life. Regardless of the status of left ventricular function (preserved, borderline, and reduced ejection fraction), the risk-adjusted analyses for the composite of mortality and rehospitalization are similar for all groups (63). Because patients with HF frequently see a worsening in their functional class and experience subsequent hospitalizations due to decompensation episodes, economic costs are significantly increasing due to frequent hospitalization and rehospitalization and the related comorbidities. Furthermore, costs related to HF place a heavy economic burden on our medical system. In a recent meta-analysis, the annual median total medical costs for HF care was $24,383 per patient; HF-specific hospitalizations contributed greatly to these costs (median, $15,879 per patient) (64). New types of devices will undoubtedly increase the economic costs of HF. New technologies require a learning curve that involves training requirements and related expenses. Additionally, device-related complications may add extra expenditure on the health care system. Currently, most devices discussed here are in the proof-of-concept phase, early feasibility studies, or first-in-human clinical trials. After early phase studies are completed, the safety of new devices must be examined in clinical trials. Safety is a key point in testing new technology, but cost-effectiveness must also be proved before implementing new devices into clinical practice. The economic burden on the US health care system is increasing (4, 65), and costs are predicted to rise to $70 billion dollars by 2030. Thus, any new medical device must be safe and cost-effective to promote benefit to the patients without superfluous costs to the health care system.

Another crucial step related to safety and efficacy is the quality of the clinical trials performed for testing new devices. Conducting a relevant clinical trial obviously involves a straightforward research question, adequate inclusion/exclusion criteria, randomization, placebos/shams, a reasonable sample size, and planning for interim analyses. However, researchers must focus on appropriate and applicable outcomes for clinical problems that will benefit patients. The use of surrogate endpoints, such as laboratory markers, might lead to inconclusive results or futile benefits that are not clinically relevant. An interim analysis focused on safety and efficacy should be performed because it allows for making evaluations and decisions during the study and confirms safety endpoints (66). Our perspective is that new trials should focus on a pragmatic approach with broad inclusion criteria and recruitment, minimal organization or resources required, flexibility to deliver the intervention, a primary outcome relevant to patients, and analyses based on intention-to-treat principles (67). Only a pragmatic trial with clinically relevant endpoints will bring tangible benefits to patients, without adding costs and unnecessary interventions.

New technologies can be challenging from the beginning because the precise indication for use and the success and failure criteria are not completely defined. The devices mentioned in this review compose a new class; therefore, specific criteria must be established to avoid their empirical evaluation. For example, a comparable situation was seen in cardiology with definitions for stent thrombosis (68) and transcatheter aortic valve replacement (69). Discrepancies in classifications from different research teams can be standardized by the Academic Research Definition. Accurate and universal definitions are important because they create a uniform understanding of the challenges associated with consistency among endpoints used in reporting clinical trial results. Using standardized clinical endpoints is beneficial as a practical language for communication among researchers, health care providers, and patients. In addition, standardized endpoints are important for regulatory agencies in approving new devices, monitoring outcomes, and dealing with healthcare reimbursement. We believe that standardized criteria are needed to avoid being too liberal or too strict in identifying indications. Likewise, medical societies should be agile enough to introduce those criteria with new technologies before their approval and integration into clinical practice.

Finally, in this review, we discuss future devices that will help treat decompensated HF. Although this device-based therapy is not intended to replace current HF treatments, each one is expected to act at specific pathways of the decompensation, thus improving patient outcomes.

## Author contributions

CO and KL made substantial contributions to literature research, conception and design, interpretation of data, and drafting the manuscript. AE and EP contributed to revising the manuscript critically for important intellectual content. All authors read and approved the final version.
